# The hypothetical periplasmic protein PA1624 from *Pseudomonas aeruginosa* folds into a unique two-domain structure

**DOI:** 10.1107/S2053230X20014612

**Published:** 2020-11-30

**Authors:** Christian G. Feiler, Manfred S. Weiss, Wulf Blankenfeldt

**Affiliations:** a Macromolecular Crystallography (HZB-MX), Helmholtz-Zentrum Berlin, Albert-Einstein-Strasse 15, D-12489 Berlin, Germany; b Structure and Function of Proteins, Helmholtz Centre for Infection Research, Inhoffenstrasse 7, D-389124 Braunschweig, Germany; c Institute for Biochemistry, Biotechnology and Bioinformatics, Technische Universität Braunschweig, Spielmannstrasse 7, D-38106 Braunschweig, Germany

**Keywords:** periplasmic proteins, unique folds, unknown function, human pathogenic bacteria, potential drug targets, *Pseudomonas aeruginosa*

## Abstract

The crystal structure of the 268-residue periplasmic protein PA1624 from the opportunistic pathogen *Pseudomonas aeruginosa* PAO1 was determined to high resolution using the Se-SAD method for initial phasing. The protein was found to be monomeric and the structure consists of two domains, domains 1 and 2, comprising residues 24–184 and 185–268, respectively. The fold of these domains could not be predicted even using state-of-the-art prediction methods, and similarity searches revealed only a very distant homology to known structures, namely to Mog1p/PsbP-like and OmpA-like proteins for the N- and C-terminal domains, respectively. Since PA1624 is only present in an important human pathogen, its unique structure and periplasmic location render it a potential drug target. Consequently, the results presented here may open new avenues for the discovery and design of antibacterial drugs.

## Abstract 2

Crystal structure analysis of the hypothetical protein PA1624 from *P. aeruginosa* reveals a novel two-domain protein architecture that is only distantly reminiscent of previously characterized structural domains.

## 1. Introduction   

As of November 2020, the Protein Data Bank (PDB; Berman *et al.*, 2000 ▸) contains more than 170 000 structural entries of biological macromolecules, of which more than 90% have been determined by X-ray crystallography. However, most of the newly deposited entries comprise folds that have already been observed in other, homologous structures. This is reflected in the notion that most of the new structures determined by X-ray crystallography are solved by molecular replacement (Long *et al.*, 2008 ▸) and also in the fact that the number of unique protein folds has not significantly increased over the last 15 years (Liu *et al.*, 2004 ▸). This suggests that the structural universe is much smaller than the sequence universe (Chothia, 1992 ▸; Levitt, 2009 ▸). Completing the catalog of protein folds invented by nature is a prerequisite for unveiling and comprehending the rules governing protein evolution, understanding the relationship between protein structure and function, and advances in *de novo* protein design. New folds may not be expected in well characterized genetic landscapes but are more likely to be found within uncharacterized gene products. Therefore, incompletely characterized genomes offer a comparatively higher chance of identifying novel and probably therapeutically interesting protein structures. One of these incompletely understood organisms is the human pathogen *Pseudomonas aeruginosa*. This Gram-negative bacterium is ubiquitous in nature (Green *et al.*, 1974 ▸) and can colonize a variety of different host organisms ranging from insects and animals to plants and mammals (D’Argenio *et al.*, 2001 ▸; Mahajan-Miklos *et al.*, 1999 ▸; Walker *et al.*, 2004 ▸). Its versatile metabolism provides a prominent evolutionary advantage, enabling *P. aeruginosa* to inhabit niches that are harmful or toxic to others (Tsuji *et al.*, 1982 ▸; Tümmler *et al.*, 2014 ▸). This makes the bacterium a severe threat to immune-compromised individuals such as AIDS patients or persons suffering from neutropenia and cystic fibrosis (CF) (Aloush *et al.*, 2006 ▸; Hidron *et al.*, 2008 ▸; Hogardt & Heesemann, 2013 ▸) and establishes it as one of the most prevalent nosocomial pathogens worldwide (Bereket *et al.*, 2012 ▸; Santajit & Indrawattana, 2016 ▸). In CF, *P. aeruginosa* evokes chronic lung infections, which is one of the main reasons for lower life expectancy, and is a significant determinant of morbidity and mortality in these patients (Kosorok *et al.*, 2001 ▸; Li *et al.*, 2005 ▸). During later stages of infection, the bacteria can disseminate via the bloodstream and affect any part of the body, making antimicrobial treatment almost impossible (van Delden, 2007 ▸; Shorr, 2009 ▸). Therefore, it is not surprising that *Pseudomonas* has been listed amongst the five top pathogens in modern times (Santajit & Indrawattana, 2016 ▸).

*P. aeruginosa* possesses a large genome that contains more than 5500 open reading frames (ORFs) in the case of the well researched strain *P. aeruginosa* PAO1. However, even though the genome sequence was completed in 2000 (Stover *et al.*, 2000 ▸), and despite the existence of a large community-based annotation effort (Winsor *et al.*, 2016 ▸), there are still more than 2200 genes predicted by bioinformatics, amounting to 35% of all predicted ORFs using *DOOR* (Mao *et al.*, 2009 ▸), that lack characterization. This uncharted territory is likely to harbor potential drug targets, and it is expected that amongst these uncharacterized genes those that encode proteins with nonpredictable folds will be highly attractive for drug development because it is less probable that they will have an overlapping function with proteins of the host organism.

Here, we describe the X-ray crystallographic structural characterization of one such gene product with unknown function and novel structure, namely the hypothetical protein PA1624 from *P. aeruginosa* PAO1.

## 2. Materials and methods   

### 2.1. Macromolecule production   

The coding region of PA1624 lacking the first 18 amino acids, representing the periplasmic localization signal, was PCR-amplified from *P. aeruginosa* PAO1 genomic DNA using the appropriate DNA primer set for cloning into *p10$*, which generates a rhinovirus 3C protease-cleavable N-terminally tagged His_6_-T7-lysozyme fusion construct, *p10$_Δ_18_PA1624* (Table 1 ▸; Bock *et al.*, 2017 ▸). The amino-acid sequence of the entire construct is mghhhhhhaenlyfqgh*TARVQFKQRESTDAIFVHCSATKPSQNVGVREIRQWHKEQGWLDVGYHFIIKRDGTVEAGRDEMAVGSHAKGYNHNSIGVCLVGGIDDKGKFDANFTPAQMQSLRSLLVTLLAKYEGAVLRAHHEVAPKACPSFDLKRWWEKNELVTSDRGHT*levlfq|gph**MADLPGSHDLDILPRFPRAEIVDFRQAPSEERIYPLGAISRISGRLRMEGEVRAEGELTALTYRLPPEHSSQEAFAAARTALLKADATPLFWCERRDCGSSSLLANAVFGNAKLYGPDEQQAYLLVRLAAPQENSLVAVYSITRGNRRAYLQAEELKADAPLAELLPSPATLLRLLKANGELTLSHVPAEPAGSWLELLVRTLRLDTGVRVELSGKHAQEWRDALRGQGVLNSRMELGQSEVEGLHLNWLR**, with lower case letters indicating the His_6_ tag and TEV cleavage site, italic letters indicating the T7-lysozyme moiety and bold letters indicating the Δ_18_PA1624 part. The symbol | denotes the cleavage site of rhinovirus 3C protease. The plasmid is available upon request.

Plasmid-harboring *Escherichia coli* BL21(DE3) cells were grown in TB medium in a 2 l fermenter at 37°C. When an OD_600_ of 2.8 was reached, the temperature was lowered to 20°C, 0.5 m*M* isopropyl β-d-1-thiogalactopyranoside (IPTG) was added and overexpression was carried out for 16 h. The harvested cells were resuspended in buffer *A* (150 m*M* Na_*x*_H_*y*_PO_4_ pH 8.0, 300 m*M* NaCl) and lysed. Cell debris and insoluble matter were separated from the soluble fraction before loading onto a precharged nickel HiTrap Chelating HP column equilibrated in buffer *A*. Nonspecifically bound proteins were removed by washing with 2% buffer *B* (buffer *A* with 500 m*M* imidazole) before a gradient elution to 100% buffer *B* was performed. 1 mg rhinovirus 3C protease (Cordingley *et al.*, 1990 ▸; Stanway *et al.*, 1984 ▸) was added to 40 mg of the fusion protein to remove the His_6_-T7-lysozyme tag during dialysis (10 kDa cutoff membrane) against buffer GF (50 m*M* HEPES, 150 m*M* NaCl pH 8.0) at 4°C overnight. The next day, the protein solution was loaded onto a HiTrap Chelating HP column precharged with nickel to separate noncleaved fusion protein from Δ_18_PA1624. The concentrated flowthrough (Macrosep Advance, 10 kDa; Pall Corporation) was applied to size-exclusion chromatography using a Superdex 26/600 S75 prep-grade column mounted on an ÄKTA system (GE Healthcare).

Seleno-l-methionine-labeled (SeMet) protein was expressed in *E. coli* Rosetta2 *pLysS* cells harboring *p10$_Δ_18_PA1624*. Briefly, a preculture was grown in LB medium supplemented with appropriate antibiotics at 37°C overnight and harvested the next day. The cells were resuspended in M9 medium, incubated for 1 h and used as an inoculum for the primary culture in prewarmed M9 medium supplemented with selective antibiotics. The cell cultures were incubated at 37°C and vigorously shaken. When the cell density reached an OD_600_ of 0.6, an amino-acid mixture inhibiting natural methionine biosynthesis was added (100 mg l^−1^ lysine, phenylalanine and threonine; 50 mg l^−1^ isoleucine, leucine and valine) and incubation was continued. The temperature was decreased to 20°C after 10 min and 0.5 m*M* IPTG and 60 mg l^−1^ seleno-l-methionine were added. The cultures were shaken for 10 h. Purification steps were performed as described for the native protein. Seleno-l-methionine incorporation was confirmed by MALDI–MS analysis.

### 2.2. Crystallization   

**Figure 1:**
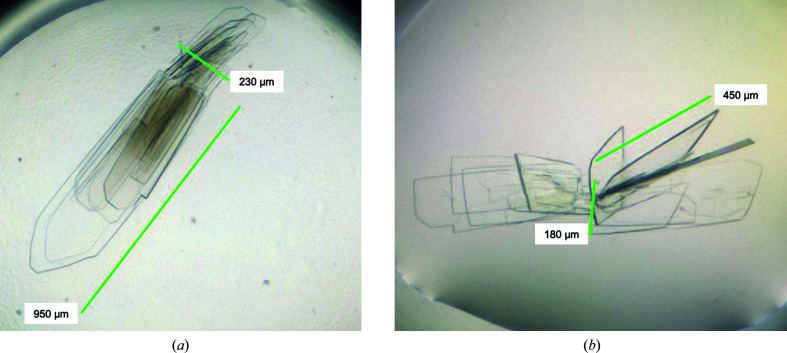
Crystals of native Δ_18_PA1624 (*a*) and selenomethionine-derivatized protein (*b*) could be obtained with slightly different shapes. The native crystals (*a*) grew as thin fragile plates with sizes of up to 950 × 250 µm. SeMet crystals (*b*) could be grown to a size of about 450 × 180 µm with a substantial third dimension.

Crystallization screening using both the native and the SeMet variant was carried out in 96-well plates. Standard sitting-drop vapor-diffusion experiments were set up at 20°C employing the commercial screens JCSG Core Suites I–IV (Qiagen). An automated liquid-dispensing robot (Phoenix, Art Robbins Instruments, USA) was employed to mix 0.1 µl concentrated protein solution (12 mg ml^−1^) with an equal volume of precipitant solution. Initial small plate-shaped crystals were obtained after five days and were refined in a grid screen using a hanging-drop vapor-diffusion setup in 24-well Linbro plates (Table 2 ▸). The final mother-liquor composition for the native crystals was 0.2 *M* sodium acetate, 0.1 *M* HEPES pH 7.7, 24.5%(*w*/*v*) PEG 4000. SeMet protein crystals were obtained using concentrated protein solution (15 mg ml^−1^) with 0.15 *M* sodium acetate, 0.1 *M* HEPES pH 7.1, 23.3%(*w*/*v*) PEG 4000. Typical protein crystals grew in thin plates to about 250 × 900 µm within ten days for native and 15 days for SeMet protein (Fig. 1 ▸). Harvested crystals were cryoprotected in mother liquor supplemented with 20%(*v*/*v*) PEG 400 and then flash-cooled in liquid nitrogen.

### 2.3. Data collection and processing   

Diffraction data were collected on beamline BL14.1 at the electron-storage ring operated by the Helmholtz-Zentrum Berlin (Mueller *et al.*, 2015 ▸). Data were collected from native crystals using a CCD detector. Data from the derivatized crystal were collected in eight 360° passes. The crystal was translated between passes. All data were indexed and integrated with *XDSAPP* (Sparta *et al.*, 2016 ▸) and scaled with *AIMLESS* (Evans & Murshudov, 2013 ▸). The 〈*I*/σ(*I*)〉 value of 2.6 in the outer resolution shell of the SeMet data indicates a non-optimal crystal-to-detector distance during data collection, suggesting that this crystal may have diffracted to an even higher resolution than the reported 1.96 Å. The calculated Matthews coefficient of 2.27 Å^3^ Da^−1^ indicated the presence of two monomers in the asymmetric unit.

All relevant data-collection and processing statistics are given in Table 3 ▸.

### 2.4. Structure solution and refinement   

Initial phases were obtained by the single-wavelength anomalous dispersion (SAD) method using the SeMet crystals. Since the protein sequence contains only two selenium-labeled methionine residues (Table 1 ▸), highly redundant data were collected. After data reduction and scaling using *XDSAPP*, structure solution was achieved with *SHELX* (Sheldrick, 2010 ▸). The anomalous signal was extracted using *SHELXC*, the substructure was determined with *SHELXD*, and *SHELXE* was used to carry out the initial model building of a polyalanine chain.

The initial model was manually corrected and adjusted in *Coot* (Emsley *et al.*, 2010 ▸). Automated refinement was carried out with the *Phenix* application *phenix.refine *(Afonine *et al.*, 2012 ▸; Liebschner *et al.*, 2019 ▸). *MolProbity* (Williams *et al.*, 2018 ▸) was used for Ramachandran analysis and evaluation of the model quality. The final model was refined to an *R*_cryst_ of 17.5% and an *R*_free_ of 23.9% against the higher resolution (1.96 Å) SeMet data set. The collected diffraction data were processed to 1.96 Å resolution. Despite a rather high signal-to-noise ratio of 2.6 in the outermost resolution bin, data beyond this resolution limit are incomplete owing to a non-optimal crystal-to-detector distance during the experiment. The Ramachandran plot shows all residues to be in the allowed region and 97% to be in the favored region. Atomic coordinates and structure factors have been deposited in the PDB with accession code 6td9. All relevant refinement and validation statistics are shown in Table 4 ▸. The secondary-structure elements were defined using *DSS* as included in the *Phenix* suite and *PSIPRED* (Buchan & Jones, 2019 ▸).

## 3. Results and discussion   

**Figure 2:**
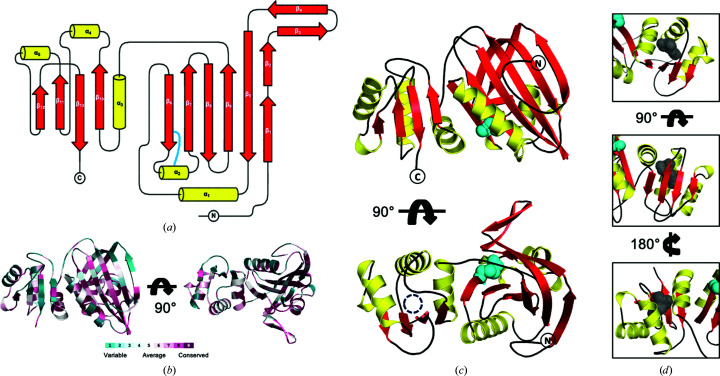
(*a*) Overall topology of Δ_18_PA1624 with α-helical elements colored yellow and β-strands red. The two domains are connected via a long linker stretching from β_9_ to α_3_. The light blue connection indicates a disulfide bridge. (*b*) The amino-acid conservation was calculated with *ConSurf* (Ashkenazy *et al.*, 2016 ▸) and plotted on the protein structure. (*c*) Ribbon representation of Δ_18_PA1624. The same coloring scheme as in (*a*) was used. The circle indicates the location of the hydrophobic cavity shown from different angles in (*d*). (*d*) The hydrophobic cavity location and its shape are depicted in three different orientations.

Here, we present the crystal structure of PA1624, a 268-amino-acid hypothetical protein from the human opportunistic pathogen *P. aeruginosa* strain PAO1 that is localized in its periplasm (Fig. 2 ▸). The protein was heterologously expressed in *E. coli* without its periplasmatic localization signal (Δ_18_PA1624). We tested several standard expression plasmids, including, for example, *pET-19*, *pMal* and *pET-28*, but using an N-terminal T7-lysozyme fusion as encoded in our self-designed *p10$* plasmid provided the best results with respect to the yield of soluble protein.

PA1624 does not display any detectable sequence homology to previously determined protein structures. Structure prediction using *Phyre*2 (Kelley *et al.*, 2015 ▸) failed to produce a reliable structure model for molecular replacement. We therefore resorted to phasing by the Se-SAD method, allowing us to determine and refine the structure to 1.96 Å resolution with *R*_cryst_ = 17.5% and *R*_free_ = 23.9%. The data collected from the native crystal were not further used for refinement and structure analysis as the diffraction data obtained from the SeMet crystals were of higher quality.

The asymmetric unit of the orthorhombic crystal form studied here contained two chains of Δ_18_PA1624, which superpose with a C^α^ r.m.s.d. of 0.5 Å, which is only slightly higher than the coordinate error. *PISA* analysis (Krissinel & Henrick, 2007 ▸) indicates that the protein is monomeric, which is in line with observations made during the course of purification by size-exclusion chromatography.

Except for a handful of flexible residues at the N-terminus, both chains could be traced with confidence. The Δ_18_PA1624 monomer has approximate dimensions of 54 × 45 × 48 Å. It folds into two distinguishable domains, comprising residues 24–184 and 185–268, as determined by *PiSQRD* (Aleksiev *et al.*, 2009 ▸). The two domains interact through a relatively small hydrophobic interface covering about 600 Å^2^. The larger domain is dominated by a six-stranded antiparallel β-sheet that is covered by one α-helix on the face that also harbors the N-terminus and by a mixed α/β structure on the other. The smaller domain features a four-stranded mixed β-sheet lined by four α-helices on the face contacting the N-terminal domain (Fig. 2 ▸ *a*). A disulfide bridge between cysteine residues 110 and 115 provides rigidity to the structures (Fig. 2 ▸ *c*).

**Figure 3:**
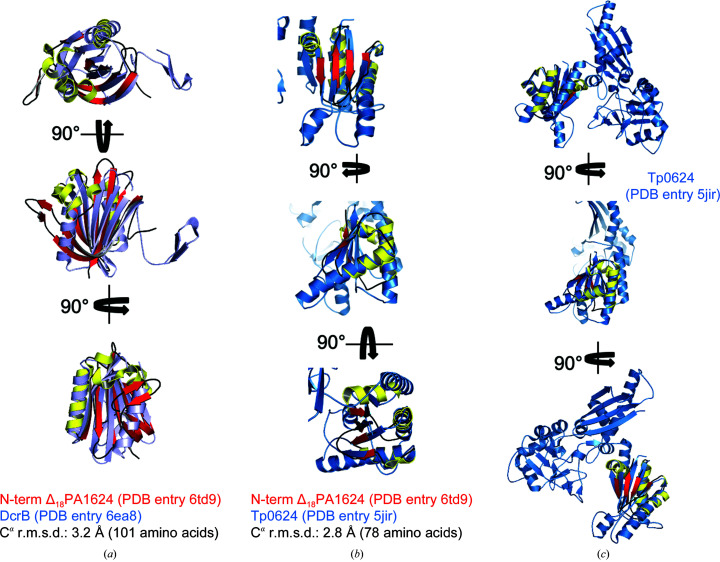
Superposition of the C- and N-terminal domains of Δ_18_PA1624 with structurally related proteins. Δ_18_PA1624 is color-coded according to its secondary-structure elements. (*a*) The N-terminal domain superposes on the full-length lipoprotein DcrB from *S. enterica*, colored light blue (PDB entry 6ea8), with a C^α^ r.m.s.d. of 3.2 Å over 101 amino acids. (*b*) The smaller C-terminal domain structurally aligned with the blue-colored domain of Tp0624 from *Treponema pallidum* with a C^α^ r.m.s.d. of 2.8 Å over 78 residues (PDB entry 5jir). (*c*) Superposition of the C-terminal domain of PA1624 with full-length Tp0624 from *T. pallidum*.

The presence of two domains in PA1624 was not anticipated, since an automated Pfam sequence analysis (Finn *et al.*, 2010 ▸) had predicted only one domain, namely a DUF4892 domain extending from positions 20 to 202. Consequently, the question arose whether the two observed domains may be related to other, already known structural building blocks or whether they indeed represent new folds. Despite no apparent sequence similarity or large conserved protein regions that could be identified (Fig. 2 ▸ *b*), we found that PA1624 is composed of two previously identified domains. For the N-terminal domain, analysis with *DALI* (Holm & Laakso, 2016 ▸) reveals distant yet significant structural homology to the DUF1795-containing lipoprotein DcrB from *Salmonella enterica* (*Z*-score 8.5; PDB entry 6e8a; Rasmussen *et al.*, 2018 ▸). The proteins align with a C^α^ r.m.s.d. of 3.2 Å over 101 residues with only 7% sequence identity, and differences are mainly owing to a β-structure insertion between β-strands 1 and 2 and additional α-helical structure between β-strands 3 and 4 in PA1624 (Fig. 3 ▸ *a*). The closest homolog of the C-terminal domain is a building block of Tp0624 from *Treponema pallidum* (*Z*-score 8.3; PDB entry 5jir; Parker *et al.*, 2016 ▸), which aligns with a C^α^ r.m.s.d. of 2.8 Å over 78 residues, displaying a sequence identity of 15% (Figs. 3 ▸ *b* and 3 ▸ *c*). The Tp0624 domain appears to be larger owing to an additional α-helix inserted between the β-strands corresponding to the third and fourth β-strand of the domain in PA1624, as well as a significantly longer α-helix following the second β-strand. Further, the first secondary-structure element of this domain in PA1624 is an α-helix, whereas Tp0624 possesses a β-strand in this position (Fig. 3 ▸ *b*, lower panel).

It is interesting to speculate about the implications for the function of PA1624 that these similarities may suggest. The previous analysis indicated that the DcrB protein is a membrane-anchored periplasmatic protein that belongs to the Mog1p/PsbP family (Rasmussen *et al.*, 2018 ▸), a group of proteins that perform diverse functions but may be associated with membrane-anchored complexes in bacteria. The identified domain of Tp0624, on the other hand, possesses strong similarities to the OmpA family, a class of proteins involved in proteoglycan binding. In comparison, this hints at a membrane-associated function within the periplasm of *P. aeruginosa* for PA1624, in line with the anticipated and the experimentally confirmed location of the protein (Imperi *et al.*, 2009 ▸). However, there are also indications that contradict such direct conclusions. Firstly, the N-terminal domain of PA1624 does not contain a cysteine at its N-terminus, as is implicated in lipid modification and membrane anchoring in DcrB. Secondly, the C-terminal OmpA-like domain lacks the conserved sequence motifs that are required for protein glycan binding in these proteins. These motifs reside in the missing secondary-structure elements mentioned above. Therefore, additional studies will be necessary to identify the function of PA1624. Towards this, it is interesting to note that the interior of the C-terminal domain of the protein is not optimally packed, leaving a cavity lined by hydrophobic residues unoccupied. This cavity may sequester a hydrophobic ligand, such as a lipidic component of the membrane (Fig. 2 ▸ *d*).

Overall, the structure of PA1624 described here confirms that the vast amount of available structural data makes it challenging to discover new protein folds, even if relationships are not apparent at the sequence level. This seems particularly true for smaller building blocks such as the two unanticipated domains found here in PA1624, since these domains will be dominated by secondary-structure elements that can only fold into a limited number of arrangements. Consequently, domains with no common ancestry will display similar structures, requiring further structure determination to reveal these relationships and inform structure-prediction programs. Therefore, we suggest that PA1624 has a novel, yet-to-be-named architectural domain arrangement.

## Supplementary Material

PDB reference: mature PA1624 from *Pseudomonas aeruginosa* PAO1, 6td9

## Figures and Tables

**Table 1:** Macromolecule-production information

Source organism	*P. aeruginosa* PAO1
DNA source	Genomic DNA
Forward primer†	AATAT*CATATG*CGAGGGTTCCTGTTGCTATC
Reverse primer‡	TTATA*CTCGAG*TCAACGCAGCCAGTTGAG
Cloning vector	*p10$*
Expression vector	*p10$*
Expression host	*E. coli* BL21(DE3) and *E. coli* Rosetta2 *pLysS*
Complete amino-acid sequence of the construct produced	GPHMADLPGSHDLDILPRFPRAEIVDFRQAPSEERIYPLGAISRISGRLRMEGEVRAEGELTALTYRLPPEHSSQEAFAAARTALLKADATPLFWCERRDCGSSSLLANAVFGNAKLYGPDEQQAYLLVRLAAPQENSLVAVYSITRGNRRAYLQAEELKADAPLAELLPSPATLLRLLKANGELTLSHVPAEPAGSWLELLVRTLRLDTGVRVELSGKHAQEWRDALRGQGVLNSRMELGQSEVEGLHLNWLR
UniProt	Q9I398_PSEAE

† The NdeI site is in italics.

‡ The XhoI site is in italics.

**Table 2:** Crystallization

Protein	SeMet Δ_18_PA1624	Native Δ_18_PA1624
Method	Vapor diffusion, hanging drop	Vapor diffusion, hanging drop
Plate type	Linbro	Linbro
Temperature (K)	293	293
Protein concentration (mg ml^−1^)	12	15
Buffer composition of protein solution	50 m*M* HEPES pH 8.0, 150 m*M* NaCl, 0.25 m*M* DTT	50 m*M* HEPES pH 8.0, 150 m*M* NaCl
Composition of reservoir solution	0.15 *M* sodium acetate, 0.1 *M* HEPES pH 7.1, 23.3% PEG 4000	0.2 *M* sodium acetate, 0.1 *M* HEPES pH 7.7, 24.5% PEG 4000
Volume and ratio of drop	2 µl, 1:1 ratio	2 µl, 1:1 ratio
Volume of reservoir (µl)	500	500

**Table 3:** Data collection and processing Values in parentheses are for the outer shell.

Protein	SeMet Δ_18_PA1624	Native Δ_18_PA1624
Diffraction source	BL14.1, BESSY II	BL14.1, BESSY II
Wavelength (Å)	0.9795	0.91840
Temperature (K)	100	100
Detector	Dectris PILATUS 6M	Rayonix 225 CCD
Crystal-to-detector distance (mm)	400	250
Rotation range per image (°)	0.1	1
Total rotation range (°)	2880	180
Exposure time per image (s)	0.1	2
Space group	*P*2_1_2_1_2_1_	*P*2_1_2_1_2_1_
*a*, *b*, *c* (Å)	53.30, 59.32, 158.54	54.42, 58.81, 163.4
α, β, γ (°)	90, 90, 90	90, 90, 90
Mosaicity (°)	0.16	0.33
Resolution range (Å)	48.0–1.96 (2.03–1.96)	30–2.40 (2.49–2.40)
Total No. of reflections	3344179 (130147)	78498 (8666)
No. of unique reflections	36764 (3576)	21179 (2202)
Completeness (%)	99.4 (98.1)	99.6 (99.8)
Anomalous completeness (%)	99.5 (96.5)	—
Multiplicity	91.0 (36.4)	3.7 (3.9)
Anomalous multiplicity	46.5 (12.2)	—
〈*I*/σ(*I*)〉	27.1 (2.6)	5.1 (1.5)
*R*_r.i.m._	0.225 (1.76)	0.261 (0.987)
*R*_p.i.m._	0.023 (0.43)	0.121 (0.414)
*R*_merge_	0.258 (2.10)	0.223 (852)
CC_1/2_	0.99 (0.56)	0.98 (0.73)
*I*/σ(*I*)_asymptotic_	24.8	10.1
Overall *B* factor from Wilson plot (Å^2^)	21.5	20.5

**Table 4:** Structure refinement Values in parentheses are for the outer shell.

Resolution range (Å)	47.49–1.96 (2.01–1.96)
Completeness (%)	99.4
σ Cutoff	0
No. of reflections, working set	36757 (2587)
No. of reflections, test set	1805 (135)
Final *R*_cryst_	0.175 (0.290)
Final *R*_free_	0.239 (0.345)
Cruickshank DPI	0.156
No. of non-H atoms	
Protein	3823
Ion	10
Ligand	29
Water	338
Total	4200
R.m.s. deviations	
Bond lengths (Å)	0.011
Angles (°)	1.24
Average *B* factors (Å^2^)	
Protein	29.3
Ion	56.2
Ligand	41.8
Water	33.7
Ramachandran plot	
Favored regions (%)	97.1
Additionally allowed (%)	2.5
Outliers (%)	0.2

## References

[bb1] Afonine, P. V., Grosse-Kunstleve, R. W., Echols, N., Headd, J. J., Moriarty, N. W., Mustyakimov, M., Terwilliger, T. C., Urzhumtsev, A., Zwart, P. H. & Adams, P. D. (2012). *Acta Cryst.* D**68**, 352–367. 10.1107/S0907444912001308 PMC3322595 22505256

[bb2] Aleksiev, T., Potestio, R., Pontiggia, F., Cozzini, S. & Micheletti, C. (2009). *Bioinformatics*, **25**, 2743–2744. 10.1093/bioinformatics/btp512 19696046

[bb3] Aloush, V., Navon-Venezia, S., Seigman-Igra, Y., Cabili, S. & Carmeli, Y. (2006). *Antimicrob. Agents Chemother.* **50**, 43–48. 10.1128/AAC.50.1.43-48.2006 PMC1346794 16377665

[bb4] Ashkenazy, H., Abadi, S., Martz, E., Chay, O., Mayrose, I., Pupko, T. & Ben-Tal, N. (2016). *Nucleic Acids Res.* **44**, W344–W350. 10.1093/nar/gkw408 PMC4987940 27166375

[bb5] Bereket, W., Hemalatha, K., Getenet, B., Wondwossen, T., Solomon, A., Zeynudin, A. & Kannan, S. (2012). *Eur. Rev. Med. Pharmacol. Sci.* **16**, 1039–1044. 22913154

[bb6] Berman, H. M., Westbrook, J., Feng, Z., Gilliland, G., Bhat, T. N., Weissig, H., Shindyalov, I. N. & Bourne, P. E. (2000). *Nucleic Acids Res.* **28**, 235–242. 10.1093/nar/28.1.235 PMC102472 10592235

[bb7] Bock, T., Luxenburger, E., Hoffmann, J., Schütza, V., Feiler, C., Müller, R. & Blankenfeldt, W. (2017). *Angew. Chem. Int. Ed.* **56**, 9986–9989. 10.1002/anie.201701992 28508504

[bb8] Buchan, D. W. A. & Jones, D. T. (2019). *Nucleic Acids Res.* **47**, W402–W407. 10.1093/nar/gkz297 PMC6602445 31251384

[bb9] Chothia, C. (1992). *Nature*, **357**, 543–544. 10.1038/357543a0 1608464

[bb10] Cordingley, M. G., Callahan, P. L., Sardana, V. V., Garsky, V. M. & Colonno, R. J. (1990). *J. Biol. Chem.* **265**, 9062–9065. 2160953

[bb11] D’Argenio, D. A., Gallagher, L. A., Berg, C. A. & Manoil, C. (2001). *J. Bacteriol.* **183**, 1466–1471. 10.1128/JB.183.4.1466-1471.2001 PMC95024 11157963

[bb12] Delden, C. van (2007). *Int. J. Antimicrob. Agents*, **30**, S71–S75. 10.1016/j.ijantimicag.2007.06.015 17698326

[bb13] Emsley, P., Lohkamp, B., Scott, W. G. & Cowtan, K. (2010). *Acta Cryst.* D**66**, 486–501. 10.1107/S0907444910007493 PMC2852313 20383002

[bb14] Evans, P. R. & Murshudov, G. N. (2013). *Acta Cryst.* D**69**, 1204–1214. 10.1107/S0907444913000061 PMC3689523 23793146

[bb15] Finn, R. D., Mistry, J., Tate, J., Coggill, P., Heger, A., Pollington, J. E., Gavin, O. L., Gunasekaran, P., Ceric, G., Forslund, K., Holm, L., Sonnhammer, E. L. L., Eddy, S. R. & Bateman, A. (2010). *Nucleic Acids Res.* **38**, D211–D222. 10.1093/nar/gkp985 PMC2808889 19920124

[bb16] Green, S. K., Schroth, M. N., Cho, J. J., Kominos, S. K. & Vitanza-Jack, V. B. (1974). *Appl. Microbiol.* **28**, 987–991. 10.1128/am.28.6.987-991.1974 PMC186868 4217591

[bb17] Hidron, A. I., Edwards, J. R., Patel, J., Horan, T. C., Sievert, D. M., Pollock, D. A. & Fridkin, S. K. (2008). *Infect. Control Hosp. Epidemiol.* **29**, 996–1011. 10.1086/591861 18947320

[bb18] Hogardt, M. & Heesemann, J. (2013). *Curr. Top. Microbiol. Immunol.* **358**, 91–118. 10.1007/82_2011_199 22311171

[bb19] Holm, L. & Laakso, L. M. (2016). *Nucleic Acids Res.* **44**, W351–W355. 10.1093/nar/gkw357 PMC4987910 27131377

[bb20] Imperi, F., Ciccosanti, F., Perdomo, A. B., Tiburzi, F., Mancone, C., Alonzi, T., Ascenzi, P., Piacentini, M., Visca, P. & Fimia, G. M. (2009). *Proteomics*, **9**, 1901–1915. 10.1002/pmic.200800618 19333994

[bb21] Kelley, L. A., Mezulis, S., Yates, C. M., Wass, M. N. & Sternberg, M. J. E. (2015). *Nat. Protoc.* **10**, 845–858. 10.1038/nprot.2015.053 PMC5298202 25950237

[bb22] Kosorok, M. R., Zeng, L., West, S. E., Rock, M. J., Splaingard, M. L., Laxova, A., Green, C. G., Collins, J. & Farrell, P. M. (2001). *Pediatr. Pulmonol.* **32**, 277–287. 10.1002/ppul.2009.abs 11568988

[bb23] Krissinel, E. & Henrick, K. (2007). *J. Mol. Biol.* **372**, 774–797. 10.1016/j.jmb.2007.05.022 17681537

[bb24] Levitt, M. (2009). *Proc. Natl Acad. Sci. USA*, **106**, 11079–11084. 10.1073/pnas.0905029106 PMC2698892 19541617

[bb25] Li, Z., Kosorok, M. R., Farrell, P. M., Laxova, A., West, S. E. H., Green, C. G., Collins, J., Rock, M. J. & Splaingard, M. L. (2005). *JAMA*, **293**, 581–588. 10.1001/jama.293.5.581 15687313

[bb99] Liebschner, D., Afonine, P. V., Baker, M. L., Bunkóczi, G., Chen, V. B., Croll, T. I., Hintze, B., Hung, L.-W., Jain, S., McCoy, A. J., Moriarty, N. W., Oeffner, R. D., Poon, B. K., Prisant, M. G., Read, R. J., Richardson, J. S., Richardson, D. C., Sammito, M. D., Sobolev, O. V., Stockwell, D. H., Terwilliger, T. C., Urzhumtsev, A. G., Videau, L. L., Williams, C. J. & Adams, P. D. (2019). *Acta Cryst.* D**75**, 861–877. 10.1107/S2059798319011471 PMC6778852 31588918

[bb26] Liu, X., Fan, K. & Wang, W. (2004). *Proteins*, **54**, 491–499. 10.1002/prot.10514 14747997

[bb27] Long, F., Vagin, A. A., Young, P. & Murshudov, G. N. (2008). *Acta Cryst.* D**64**, 125–132. 10.1107/S0907444907050172 PMC2394813 18094476

[bb28] Mahajan-Miklos, S., Tan, M.-W., Rahme, L. G. & Ausubel, F. M. (1999). *Cell*, **96**, 47–56. 10.1016/s0092-8674(00)80958-7 9989496

[bb29] Mao, F., Dam, P., Chou, J., Olman, V. & Xu, Y. (2009). *Nucleic Acids Res.* **37**, D459–D463. 10.1093/nar/gkn757 PMC2686520 18988623

[bb30] Mueller, U., Förster, R., Hellmig, M., Huschmann, F. U., Kastner, A., Malecki, P., Pühringer, S., Röwer, M., Sparta, K., Steffien, M., Ühlein, M., Wilk, P. & Weiss, M. S. (2015). *Eur. Phys. J. Plus*, **130**, 141.

[bb31] Parker, M. L., Houston, S., Wetherell, C., Cameron, C. E. & Boulanger, M. J. (2016). *PLoS One*, **11**, e0166274. 10.1371/journal.pone.0166274 PMC5104382 27832149

[bb32] Rasmussen, D. M., Soens, R. W., Davie, T. J., Vaneerd, C. K., Bhattacharyya, B. & May, J. F. (2018). *J. Struct. Biol.* **204**, 513–518. 10.1016/j.jsb.2018.10.005 PMC9976613 30339832

[bb33] Santajit, S. & Indrawattana, N. (2016). *Biomed Res. Int.* **2016**, 2475067. 10.1155/2016/2475067 PMC4871955 27274985

[bb34] Sheldrick, G. M. (2010). *Acta Cryst.* D**66**, 479–485. 10.1107/S0907444909038360 PMC2852312 20383001

[bb35] Shorr, A. F. (2009). *Crit. Care Med.* **37**, 1463–1469. 10.1097/CCM.0b013e31819ced02 19242341

[bb36] Sparta, K. M., Krug, M., Heinemann, U., Mueller, U. & Weiss, M. S. (2016). *J. Appl. Cryst.* **49**, 1085–1092.

[bb37] Stanway, G., Hughes, P. J., Mountford, R. C., Minor, P. D. & Almond, J. W. (1984). *Nucleic Acids Res.* **12**, 7859–7875. 10.1093/nar/12.20.7859 PMC320205 6093056

[bb38] Stover, C. K., Pham, X. Q., Erwin, A. L., Mizoguchi, S. D., Warrener, P., Hickey, M. J., Brinkman, F. S., Hufnagle, W. O., Kowalik, D. J., Lagrou, M., Garber, R. L., Goltry, L., Tolentino, E., Westbrock-Wadman, S., Yuan, Y., Brody, L. L., Coulter, S. N., Folger, K. R., Kas, A., Larbig, K., Lim, R., Smith, K., Spencer, D., Wong, G. K., Wu, Z., Paulsen, I. T., Reizer, J., Saier, M. H., Hancock, R. E., Lory, S. & Olson, M. V. (2000). *Nature*, **406**, 959–964. 10.1038/35023079 10984043

[bb39] Tsuji, A., Kaneko, Y., Takahashi, K., Ogawa, M. & Goto, S. (1982). *Microbiol. Immunol.* **26**, 15–24. 10.1111/j.1348-0421.1982.tb00149.x 7087800

[bb40] Tümmler, B., Wiehlmann, L., Klockgether, J. & Cramer, N. (2014). *F1000Prime Rep.* **6**, 9. 10.12703/P6-9 PMC3913036 24592321

[bb41] Walker, T. S., Bais, H. P., Déziel, E., Schweizer, H. P., Rahme, L. G., Fall, R. & Vivanco, J. M. (2004). *Plant Physiol.* **134**, 320–331. 10.1104/pp.103.027888 PMC316311 14701912

[bb42] Williams, C. J., Headd, J. J., Moriarty, N. W., Prisant, M. G., Videau, L. L., Deis, L. N., Verma, V., Keedy, D. A., Hintze, B. J., Chen, V. B., Jain, S., Lewis, S. M., Arendall, W. B., Snoeyink, J., Adams, P. D., Lovell, S. C., Richardson, J. S. & Richardson, D. C. (2018). *Protein Sci.* **27**, 293–315. 10.1002/pro.3330 PMC5734394 29067766

[bb43] Winsor, G. L., Griffiths, E. J., Lo, R., Dhillon, B. K., Shay, J. A. & Brinkman, F. S. L. (2016). *Nucleic Acids Res.* **44**, D646–D653. 10.1093/nar/gkv1227 PMC4702867 26578582

